# Attributing Cause of Death for Patients with *Clostridium difficile* Infection

**DOI:** 10.3201/eid1810.120202

**Published:** 2012-10

**Authors:** Rodica Gilca, Charles Frenette, Nathanaëlle Thériault, Élise Fortin, Jasmin Villeneuve

**Affiliations:** Institut National de Santé Publique du Québec, Québec City, Québec, Canada (R. Gilca, E. Fortin);; Québec University Hospital Centre, Québec City (R. Gilca);; Laval University, Québec City (R. Gilca);; McGill University Health Center, Montreal, Québec, Canada (C. Frenette, E. Fortin);; and Direction Régionale de Santé Publique, Québec City (N. Thériault, J. Villeneuve)

**Keywords:** Clostridium difficile, death, causality, bacteria, Canada

**To the Editor:** Hota et al. report that for deceased patients who had *Clostridium difficile* infection (CDI), agreement is poor between causes of death reported on death certificates and those categorized by a review panel (*1*). Our data support the difficulty of attributing cause of death for patients with CDI.

In 2004 in Quebec, Canada, a mandatory CDI surveillance program was implemented. Deaths that occurred within 30 days after CDI diagnosis were classified as 1) directly attributable to CDI (e.g., toxic megacolon, septic shock), 2) having a CDI contribution (e.g., acute decompensation of chronic heart failure), or 3) unrelated to CDI (e.g., terminal cancer) (*2*). To determine accuracy of the surveillance classifications, we compared cause-of-death classification of 22 deceased CDI patients reported to surveillance by 1 hospital in 2007 with causes of death reported by 13 external reviewers who examined summaries of medical files of the deceased patients. Reviewers were 11 infectious disease and 2 public health physicians involved with CDI surveillance at their respective hospitals but not this hospital. The median (minimal, maximal) κ statistics for comparison of external reviews with surveillance classification were 0.495 (0.252, 0.607) for directly attributable, 0.182 (−0.091, 0.182) for contributed, and 0.321 (0.124, 0.614) for unrelated. Comparison within external reviewers yielded 0.697 (0.394, 1.0), 0.233 (−0.294, 0.703), and 0.542 (0.154, 0.909), respectively. Complete agreement was found for only 6 cases (4 directly attributable and 2 unrelated) (Figure).

**Figure:**
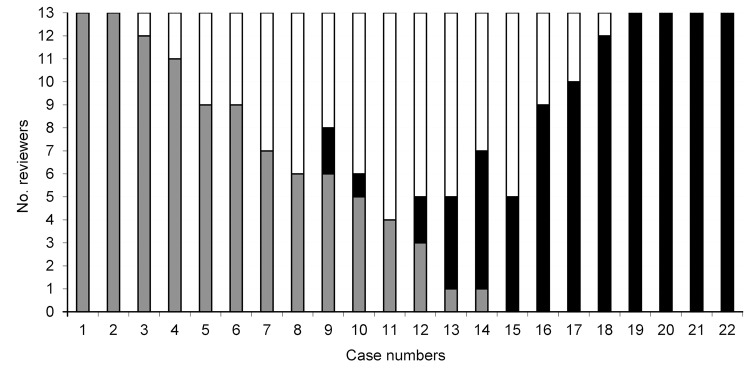
Classification of cause of death among 22 patients with *Clostridium difficile* infection (CDI), by 13 external reviewers, Quebec, Canada, 2007. Bars indicate the number of reviewers who assigned each category. Gray bars indicate that CDI was unrelated to death, white bars indicate that CDI contributed to death, and black bars indicate that death was directly attributable to CDI.

Variation among reviewers suggested that categorizations reported to surveillance were inaccurate. Number of deaths among patients with CDI, regardless of the cause of death, seemed to better indicate CDI severity. Since 2008, only the crude numbers of deaths, not subjected to individual interpretation, have been reported to surveillance. A questionnaire addressing concurrent medical conditions, prognosis, level of care, and circumstances of death is being implemented in Quebec hospitals participating in CDI surveillance and should help determine the role of CDI in deaths.
